# A Network Perspective on the Comorbidity of Personality Disorders and Mental Disorders: An Illustration of Depression and Borderline Personality Disorder

**DOI:** 10.3389/fpsyg.2021.680805

**Published:** 2021-07-06

**Authors:** Annemarie C.J. Köhne, Adela-Maria Isvoranu

**Affiliations:** ^1^Department of Psychiatry, University Medical Center Utrecht, Utrecht, Netherlands; ^2^Academic Medical Center, Amsterdam, Netherlands; ^3^Department of Psychology, University of Amsterdam, Amsterdam, Netherlands

**Keywords:** network theory, comorbidity, personality disorder, depression, complexity

## Abstract

The comorbidity of personality disorders and mental disorders is commonly understood through three types of theoretical models: either a) personality disorders precede mental disorders, b) mental disorders precede personality disorders, c) mental disorders and personality disorders share common etiological grounds. Although these hypotheses differ with respect to their idea of causal direction, they all imply a latent variable perspective, in which it is assumed that either personality and mental disorders are latent variables that have certain causal relations [models a) and b)]; or that, as in model c), the common etiology is in fact a latent variable that causes symptomatology of both personality and mental disorders. We aim to provide another perspective on the comorbidity between personality and mental disorders, namely a network perspective. To this end, we investigated Major Depression (MD) and Borderline Personality Disorder (BPD) and hypothesized that symptoms of BPD and MD could interact with one another rather than being caused by a latent variable (e.g., identity diffusion → unstable relationships → depressed mood). To illustrate this theoretical network conceptualization of the comorbidity of BPD and MD we analyzed a cross-sectional clinical dataset of 376 patients who were asked to complete the Structured Clinical Interview for DSM-IV Axis II Disorders and the Beck Depression Inventory II. The results identify direct associations between symptoms of MD and BPD. If we take the links in this empirical network to be ‘substantive', this suggests a radical shift of our current conceptualization of the comorbidity of mental disorders and personality disorders.

## Introduction

The ontological status of psychological disorders and comorbidity between them is at the heart of most controversy and debate within the fields of clinical psychology and psychiatry: what are mental disorders exactly? And, as a consequence, what is comorbidity, and how does it arise? Since the way we understand psychological disorders and their comorbidity determines our outlook on both research and treatment (e.g., if we hypothesize that major depression is a brain disorder, research would dedicate itself to finding the responsible brain anomaly, while treatment would focus on eradicating that anomaly), it is of the utmost importance that our ontological hypotheses are roughly correct. For decades, a latent variable perspective dominated our understanding on psychopathological phenomena, but in recent years, another perspective–the network perspective– has offered an alternative understanding of how psychological disorders and comorbidity between them may arise (Cramer et al., 2010, 2012; Borsboom, 2017). In the present paper, we outline this network perspective and provide an empirical illustration for two highly comorbid disorders: a personality disorder (borderline personality disorder; henceforth: BPD) and a common mental disorder (major depression; henceforth: MD).

## A Latent Variable Perspective on MD-BPD Comorbidity

Three latent variable perspectives purport to explain the comorbidity between BPD and MD: a) BPD precedes MD (e.g., “the vulnerability hypothesis”; “the pathoplasticity hypothesis”) or b) MD precedes BPD (e.g., “the scar hypothesis”; “the complication hypothesis”) or c) MD and BPD share common etiological grounds (e.g., Clark and Mineka, 1994; Eurelings-Bontekoe et al., 2009; Klein et al., 2011; De Bolle et al., 2012). Although these three types of theoretical models differ with respect to their idea of causal direction, they all imply a latent variable perspective in which it is assumed that either BPD or MD are latent variables that have certain causal relations [models a) and b)]; or that, as in model c), the common etiology is in fact a latent variable that causes the symptomatology of both BPD and MD. However, there is hardly any evidence of the existence of a latent disease (Zachar and Kendler, 2007; Kendler, 2012; McNally et al., 2015; Borsboom, 2017; Borsboom et al., 2019). The inability to prove the existence of such disease entities (e.g., a certain genetical make-up that causes symptoms of MD) raises the question if other theoretical and methodological approaches might provide a better understanding of the relationship between MD and BPD.

In an attempt to address this question, the current paper will i) outline the recent psychometric network perspective on the comorbidity of MD and BPD, ii) provide an empirical illustration of a comorbidity network of symptoms of MD and BPD and lastly, iii) argue embracing novel research perspectives may be beneficial and aid progress in the field of mental health, while also providing some recommendation for further research.

## A Network Perspective on MD-BPD Comorbidity

The *network theory* provides us with a different theoretical approach than current approaches outlined above. The first and central principle of the network theory is that mental disorders are complex systems: “[They] are multifactorial in constitution, aetiology, and causal background” (Borsboom, 2017, p. 7). The network theory holds that the relation between symptoms and diagnosis is mereological: symptoms are the particulars that are the diagnosis instead of that symptoms reflect an underlying disorder (Borsboom and Cramer, 2013). In analogy; jackals, coyotes, dogs, foxes and wolves are not caused by the canine family, they are the parts that constitute it. Within the network approach, disorders are conceptualized as clusters of directly related symptoms. Viewing and understanding comorbidity from a network perspective means that comorbidity arises from direct relations between symptoms of multiple disorders (Cramer et al., 2010). From the network perspective, the co-occurrence of two disorders is conceptualized as a symptom-to-symptom association between two disorders. For example, symptoms of a panic disorder and agoraphobia may hang together in the following way: panic attack in public space → worry about new panic attack in public space → avoid public spaces → avoid coming out of the house (Borsboom, 2008). And the symptoms of MD and generalized anxiety disorder may hang together in the following way: sleeping problems → fatigue and agitation → loss of interest in activities and people (Cramer et al., 2010).

In response to the network theory, statistical tools–now known as the field of network psychometrics (Epskamp et al., 2018b; Marsman et al., 2018)–have been developed, which allow investigating these particular associations between symptoms (Epskamp and Fried, 2018). Psychometric networks are constructed using nodes and edges. Nodes represent entities such as people, cities, theoretical constructs or, in this case, symptoms. Edges represent some sort of connection or relation between a pair of nodes like friendship, interaction, distance, or in this case, a statistical association. The activation of one node can transmit to other nodes, which in effect can alter the homeostasis of a network. A symptom like sleeping problems may activate other nodes like feeling tired, worry or difficulty concentrating. This spread of symptoms may alter the homeostasis from mental health to ill-health (depression). Symptoms that connect two mental disorders are often addressed as bridge nodes (Cramer et al., 2010). It has been argued that identifying and targeting these bridge symptoms therapeutically, may prevent comorbidity in which one disorder activates (bridges over to) the next disorder.

**Figure 1:**
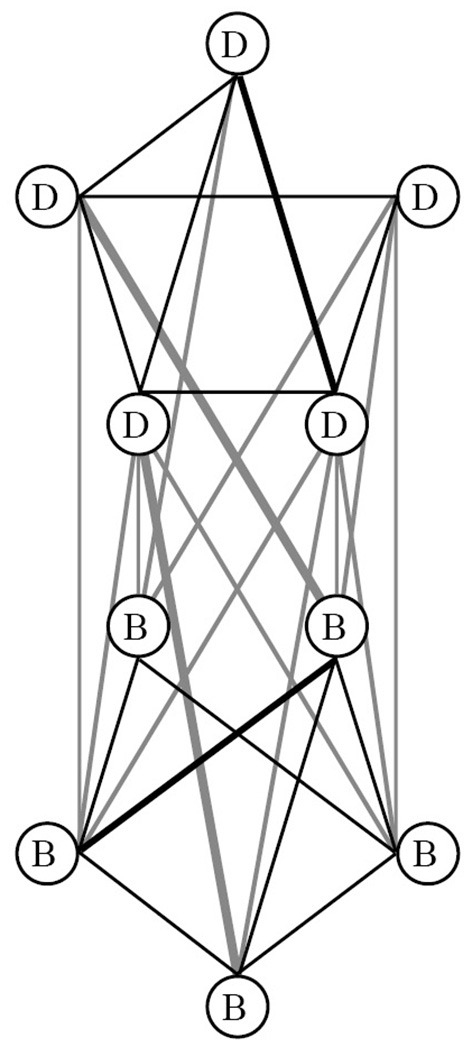
An illustration of the network approach on the comorbidity of depression symptoms (D) and borderline personality disorder symptoms (B). The network model hypothesizes associations between the symptoms of borderline personality disorder (B) and major depressive disorders (D). Some relations between symptoms are stronger than others [illustrated by the thickness of some lines (edges)].

In the last decade, numerous studies investigated the network structures of a wide variety of mental disorders (Robinaugh et al., 2020), highlighting numerous associations between symptoms. Studies into the network architectures of personality have grown prominent as well (Cramer et al., 2012; Richetin et al., 2017). Yet, the investigation of joint network structures of mental disorders and personality disorders, to our best knowledge, has not yet been carried out. In an effort to illustrate the network perspective on the comorbidity of mental disorders and personality disorders, we aimed to investigate the extent to which symptom components of BPD are related to symptoms of MD, in a clinical sample of patients suffering from co- and multi-morbidity. In the network model, the existence of a latent disorder remains an unproven hypothesis and we therefore hypothesize the presence of direct associations between the symptoms of BPD and MD (see Figure 1).

### The Comorbidity of MD and BPD

We focus on these two mental disorders because they are well-described, extensively studied, belong to the most prevalent of personality disorders and mental disorders, are known for their high disease burden, and are accompanied by high rates of mortality by suicide (e.g., Grant et al., 2008; Kessler and Bromet, 2013). Depression is projected to become the second cause of disability worldwide by the World Health Organization (e.g. Kessler and Bromet, 2013) and is characterized by loss of interest in activities and the presence of a sad, empty, or irritable mood, accompanied by somatic and cognitive changes that significantly affect the individual's capacity to function (American Psychiatric Association, 2013). Lifetime prevalence of MD in adults varies between 16% and 25% (e.g., Patten, 2009). It is a highly recurrent mental disorder in which comorbidity with other mental disorders and substance use disorders is norm rather than exception. The disease burden of MD is very high and from an economic and societal perspective, depression is perhaps one of the costliest mental disorders as well. The disease burden of people with BPD is considerably high as well; it is associated with severe functional impairment and substantial treatment utilization (e.g., Grant et al., 2008). BPD is characterized by a pervasive, persistent and pathological pattern of difficulties in the field of emotion regulation, impulse control and instability in relationships and self-image (Skodol et al., 2002; American Psychiatric Association, 2013). In the United States the lifetime prevalence of BPD is about 6% (Grant et al., 2008). Of the people diagnosed with BPD, about 85% meet criteria for one or more mental disorders (common are trauma, addiction and mood disorders) and about 74% also meet the criteria of another personality disorder (Zanarini et al., 1998; Coid, 2003; Lenzenweger et al., 2007; Grant et al., 2008). Rates of any 12-month mood disorder or MD among respondents with lifetime BPD are about 51% and 31%, respectively. And the prevalence of BPD among respondents with mood disorders or MD are about 29% and 19%, respectively (Grant et al., 2008).

## An Empirical Illustration of a Comorbidity Network of Symptoms of MD and BPD

### Participants

To illustrate a comorbidity network of symptoms of MD and BPD we analyzed data from 376 patients from the Dutch Healthcare institution *GGZ Momentum* who all had been hospitalized for 9 weeks of clinical treatment (123 women, 253 men, *M age* = 34.27, range: 18–72, *SD* = 9.90). The primary DSM-classification for admission was an addiction of some sort and all patients suffered from co- and multi-morbidity. The advantage of choosing a population with this primary diagnosis is that we could control for substance abuse in our measurements (strictly no substance use was allowed during clinical admission) and that we could control for addiction in the sample since about 78% of adults with BPD also develop a substance related disorder or addiction at some time in their lives (Kienast et al., 2014). Exclusion criteria for treatment were i) a history of severe head injury, ii) intellectual disabilities (IQ < 75), iii) a Body Mass Index below 17 and/or iv) a heroin and/or opiate addiction. Inclusion criteria for the diagnostic screening assessment of these hospitalized patients were i) a 6 weeks period of abstinence, ii) an age between 18 and 75 years, iii) the absence of cognitive impairment and iv) the absence of a current manic or psychotic episode. The study was run after review and approval from the local Institutional Review Board of the Health Care Institution *GGZ Momentum*. All patients had the adequate cognitive and language capabilities to read and process all information and give their written informed consent.

### Procedure

During the hospitalization of nine weeks, patients were asked to complete a diagnostic screening assessment after six weeks of abstinence. This was done to facilitate and objectify descriptive diagnostics. At the start of the diagnostic assessment, participants signed an informed consent and agreed to the examination of their clinical file. All patients were given the opportunity to ask any further questions about the research project. The participants were assessed using four screening instruments; the Symptom Checklist (SCL-90; Derogatis and Unger, 2010), the Beck Depression Inventory - Second Edition (BDI-II; Beck et al., 1996), the Utrecht Coping List (UCL; Schreurs et al., 1993) and the Dutch questionnaire version of the Structured Clinical Interview for DSM-IV Axis II Personality Disorders (SCID-II; Weertman et al., 2003). At the request of the primary practitioner, additional diagnostic screening instruments were added to personalize, broaden or deepen the diagnostic assessment. The participants from the sample completed all of these measures, yet we did not use them in the analyses and thus do not mention them further in this contribution. The screenings were administered by psychologists (in training) who were supervised and trained by a registered Health Care Psychologist who is also the corresponding author of this paper. In addition, all patients received a DSM-classification from their primary practitioner (registered clinical psychologist or psychiatrist) after the diagnostic screening assessment (week 6–7) or at the end of their treatment (week 9–10). Data were gathered from clinical records after clinical discharge.

### Materials

Symptoms of BPD were measured using the Dutch-language self-report version of the Structured Clinical Interview for DSM-IV Axis II Personality Disorders (SCID-II; Weertman et al., 2003). The SCID-II measures all DSM-IV criteria of all personality disorders and is composed of 119 items, of which 15 assess the 9 BPD criteria (e.g., fear of abandonment, identity diffusion). The items of the SCID-II have a “yes-no” format. The cut-off value of BPD is 5 out of 9 criteria, with a sensitivity between 87% and 100% and specificity between 52.2% and 75% (e.g., Jacobsberg et al., 1995; Hilderson et al., 2011). The SCID-II is a widely used screening instrument with good internal consistency (α = 0.71 /0.94; Maffei et al., 1997) and a moderately good test-retest reliability (*kappa* = 0.63; Weertman et al., 2003). The competitive validity is unknown due to the lack of a gold standard (First et al., 1997). Some of the 9 criteria of BPD were measured by more than one SCID-II item. The BPD criterion “identity diffusion” is measured by four SCID-II items, the criterion “anger” by three SCID-II items and the BPD criterion “self-harm/suicidality” is measured by two SCID-II items. All other BPD criteria are measured by only one item. We scored the BPD criterion positive if one or more of the corresponding items was scored positive.

The Beck Depression Inventory-II (BDI-II; Beck et al., 1996; Dutch translation by van der Does, 2002) was used to screen for symptoms of depression. The BDI-II is one of the most widely used and empirically validated questionnaires for screening depression. The BDI-II is a self-report questionnaire measuring the symptoms (e.g., sadness, low energy) and severity (range: mild, moderate, severe) of depression with 21 items. Participants choose the statement that has been most representative of them in the past 7 days. Each item is rated on a 4-point Likert-scale ranging from 0 to 3. The reliability and validity of the BDI- II have been well-established (Beck et al., 1996). To acquire higher statistical power to render the network-analysis, we only used the BDI-II items that matched best with the DSM-criteria of MD. We made sure to include variables that measure different DSM criteria instead of including similar variables that aim to measure the same DSM criterion. In order to do this, we critically assessed and compared all BDI-II items with the DSM-IV criteria of MD and selected 10 items that fitted and matched best with the different DSM-criteria of MD. Thus, our analysis focused on core symptoms of depression and borderline personality disorder.

### Network Estimation

We constructed a network where each of the items is represented as a node and an edge between two nodes (i.e., items) denotes the partial correlation between them, after controlling for all other items in the network. The nodes in the network model represent the symptom criteria of MD and BPD and the edges represent the relationships between the symptom criteria (weighted and undirected associations). This comorbidity network structure of BPD and MD was derived using the “Graphical Least Absolute Shrinkage and Selection Operator” (glasso) method that estimates a penalized maximum likelihood solution based on the Extended Bayesian Information Criterion (EBIC; Chen and Chen, 2008). In this way, we were able to estimate a parsimonious (“sparse”) network that accounts for most variance with the fewest number of edges. We used the EBICglasso regularization method, as implemented in the R software package *bootnet* version 1.4. (Epskamp et al., 2018a) and visualized the network structure using the R software package *qgraph* version 1.6.5. (Epskamp et al., 2012). The EBICglasso network estimation requires a tuning parameter, which was set to a default value of 0.5.

The placement of the nodes within the network is based on the Fruchterman-Reingold algorithm, whereby nodes with strong associations are placed more in the center of the network and nodes with weaker associations in the periphery of the network (Fruchterman and Reingold, 1991). Positive (negative) associations between symptom criteria (nodes) are represented as blue (red) edges. The thickness of the edges represents the strength of the association. The thicker the edges between the nodes, the higher the partial correlation.

### Robustness

A robustness analysis was carried out to investigate the accuracy and stability of the comorbidity network, using the R package *bootnet* version 1.4 (Epskamp et al., 2018a). We estimated the accuracy of the edge weights by performing a non-parametric bootstrap analysis to calculate the 95% bootstrapped confidence intervals (CIs) for the edges by sampling the data 1000 times (including replacement). Thereby a distribution of edge weights was generated.

### Results

In total, 376 patients were included in the analyses. There were no missing data. Of the participating patients, 67% were male and 33% were female. Table 1 presents the demographic and diagnostic (DSM) characteristics of the sample.

**Table 1:** Demographic and diagnostic (DSM) characteristics of participating patients: n (%).

**Variable**	**Total**	**(*n* = 376)**
Demographics		
Male	253	(67.29%)
Female	123	(32.71%)
DSM classification		
Any mental disorder (Axis I)	373	(99.20%)
Any personality disorder (Axis II)	250	(66.49%)
Any mental disorder and any personality disorder	249	(66.22%)
(Traits of) borderline personality disorder	80	(21.28%)
Other/unspecified (traits of) personality disorder	207	(55.05%)
Major depressive disorder	79	(21.01%)
Any mood disorder	104	(27.66%)
Major depressive disorder and (traits of) borderline personality disorder	20	(5.32%)
Any mood disorder and (traits of) borderline personality disorder	25	(6.65%)

### The Comorbidity Network of MD and BPD Symptoms

**Figure 2:**
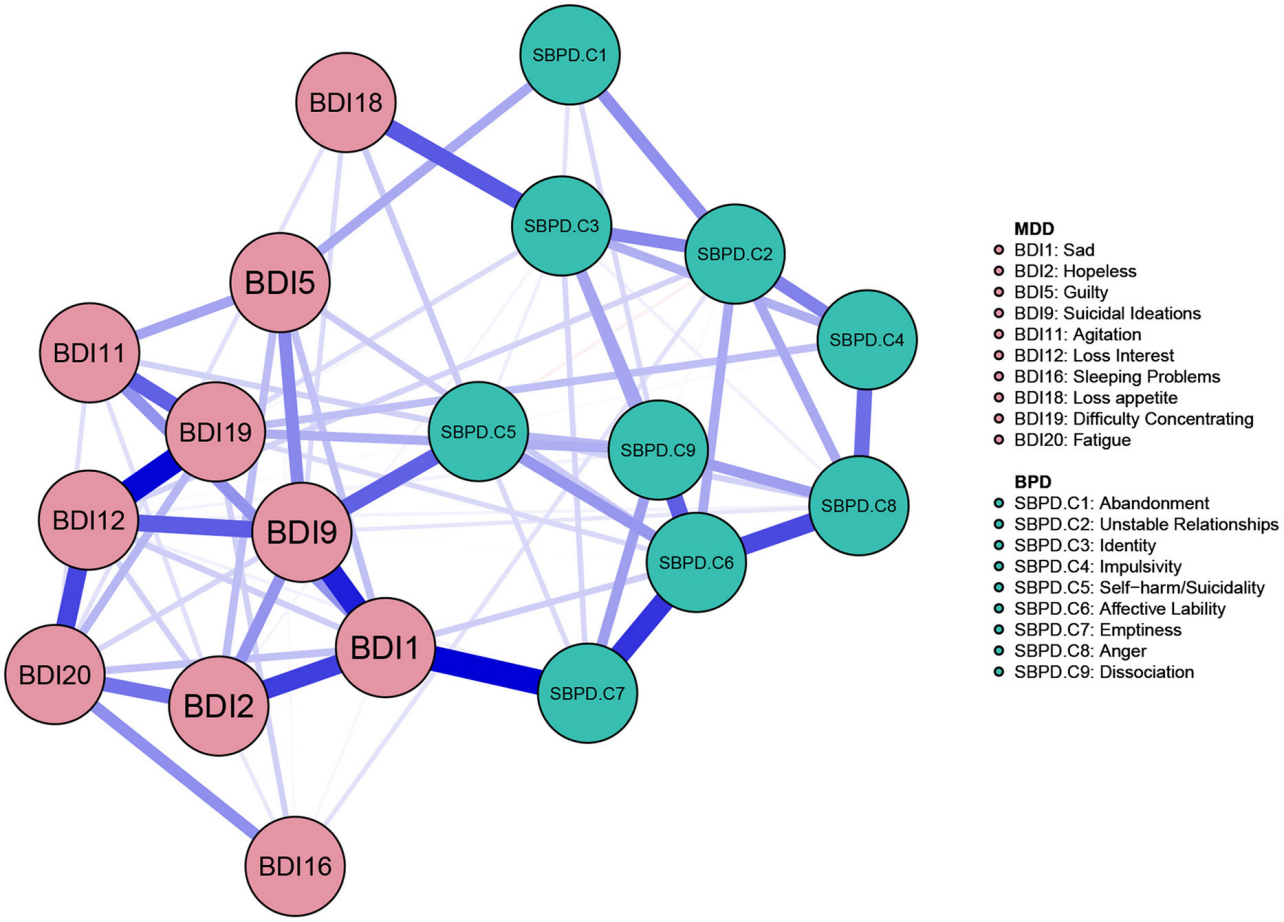
Network of the BPD and MD symptoms. Symptom groups are differentiated by color. Each edge within the network corresponds to a regularized partial correlation between 2 individual symptoms, after controlling for all other symptoms in the network. The thickness of an edge represents the absolute magnitude of the correlation (thicker edges represent stronger links), whereas the color of the edge indicates the size of the correlation (blue for positive links and red for negative links).

The resulting network (see Figure 2) illustrates the relationships between the BPD symptoms and the MD symptoms. An examination of the comorbidity network shows direct associations between the symptoms of MD and BPD, and shows that almost all connections are positive.

The strongest positive link in this comorbidity network is the one between MD sadness (BDI 1) and BPD emptiness (SBPD. C7). Furthermore, MD “suicidal ideations” (BDI 9) is positively associated to BPD “self-harm/suicidality” (SBPD. C5). MD “loss of appetite” (BDI 18) is positively associated to BPD “identity” (SBPD. C3).

Of note, likely due to the limited amount of data available, the robustness analyses of the overall network structure identified overall low stability (see Supplementary Figures 1, 2). Specifically, the sample values lie within the bootstrapped confidence intervals and the bootstrap mean values are generally aligned with the sample values, however, and of note, the bootstrapped CIs are very wide, ranging from positive to negative values for many of the edges. We have thus been conservative with the interpretation of individual connections between the nodes, and especially with the presence and strength of the smaller edges in the network structure. We based our results on the edge significance different test (see Supplementary Figure 2) when discussing edges that stand out in the comorbidity network. Further, we performed a supplementary robustness check on the network structure using Spearman correlations and retrieved a very similar result (see Supplementary Figure 3).

## Discussion

This contribution aims to emphasize the importance of embracing novel methodological and theoretical frameworks in the field of comorbidity research and brings as an exemplification the first network-analysis of the associations between symptoms of MD and BPD. In sum, the analyses show associations within and between the symptom clusters of MD and BPD. If we take the links in the network to be “substantive”, it suggests a radical shift of our current conceptualization of the comorbidity between mental disorders and personality disorders.

The strongest positive link of this comorbidity network is the one between MD sadness (BDI 1) and BPD emptiness (SBPD. C7). One could argue that these two items measure a similar construct since patients that report severe depressive symptomatology may also report feelings of emptiness (Sharma and Copeland, 1989). If we, on the other hand, consider these two symptoms to represent different constructs belonging to different disorders, we may be able to formulate some new hypotheses. Previous studies show the BPD criterion of emptiness to be a robust predictor of depression (e.g. see Klonsky, 2008) and other studies reasoned that emptiness is a distinct characteristic in BPD patients with MD (e.g. Silk, 2010).

If we form a hypothesis on this particular link with the empirically derived network model in mind, we may suppose that overwhelming feelings of sadness may (unconsciously) lead people to shut out their emotions which leads to feelings of emptiness. This emptiness may sadden patients which in effect nullifies the emptiness and so forth. This hypothesis implies that the problem that manifests itself in this particular link, is a troubled emotion-regulation (overwhelm <-> detachment). In future research, it may be interesting to study whether therapies that address emotion regulation may not only help overcome BPD symptoms, but also have an indirect positive effect on symptoms of depression if this kind of comorbidity is present. Indeed, emotion-regulation is found to be a transdiagnostic factor (e.g., see Heycop ten Dam et al., 2014; Sloan et al., 2017). This may indicate a need to develop more integrated and unified treatments that target emotion regulation for individuals who present with depression and borderline personality disorder.

If we try to translate these outcomes to an individual patient, more dept into the clinical implications of this contribution may become apparent. For example, Emma experiences instability in her emotions and becomes very angry from time to time. Because of this, she experiences difficulties in relationships and as a result she often feels unconnected and empty. This makes her sad and she often experiences hopelessness. Emma feels guilty and ashamed of herself and hides these feelings to everyone in her surroundings which in effect augments her feelings of unconnectedness, emptiness and depression. Contrary to common sense, it is innovative to understand these different symptoms as locally dependent. If the symptoms of these two disorders are meaningfully connected, it may be relevant to identify and target the important links therapeutically by which we may prevent comorbidity in which one disorder activates the next disorder. Whether this will lead to more effective treatment options for this type of comorbid psychopathology, remains an interesting empirical question for future research.

With these findings the dominant latent variable perspective that is embedded in the common types of theoretical models on the comorbidity of personality disorders and mental disorders may have to make room for an alternative understanding of how psychological disorders and the comorbidity between them arises. The comorbidity of personality disorders and mental disorders may arise from direct relations between symptoms of the two psychological disorders. We may not need latent variables or diseases to explain this type of comorbidity. The point here is not to explicate an anti-realist stance with regards to latent variables, but to explicate an anti-essentialist stance in understanding and explaining mental disorders. An anti-essentialist stance objects the view that clusters of symptoms reflect and should be explained by an underlying disorder essence. The idea that there exist meaningful links between the constructs of personality disorder and mental disorder corresponds with a psychodynamic understanding of psychopathology. The second edition of the Psychodynamic Diagnostic Manual (PDM-2; Lingiardi and McWilliams, 2015) explicates the idea that there exists a strong connection between a patient's personality and symptom patterns, suggesting that symptoms can be better understood against the background of the overall personality functioning (Hilsenroth et al., 2018).

The current contribution strives to bring a relatively non-essentialist and bottom-up conception of psychopathology. Nonetheless, by using DSM symptom components within the analyses, we still stick to a somewhat reductionist and essentialist frame of thought. Although the current study explicitly refrains from the reification of DSM syndromes and refrains from essentialist explanations, due to conventional measures is restricted to using DSM symptom components in the analysis. Thereby, the implicit assumption is that mental disorders can be ontologically described in terms of DSM symptoms. This fundamental presumption with regards to the very building blocks of psychopathology does however seem to be a reduction based on an essentialist frame of thought. To be truly non-reductionist at heart, means to transcend a mere (DSM) symptom-based account of mental disorders when building these kind of comorbidity networks in future research. To fully grasp the mechanism of a patient's mental disorder, we may need other building blocks than symptom components. If we stick to the basic idea of a multi-factorial constitution, it means we have to take into account the interacting layers of the neuro-biological, psycho-experiential and socio-cultural when understanding the causes and mechanisms of mental disorder.

Next to these more fundamental issues, several methodological limitations of the current study should be taken into consideration. First, some links may be positive because they (partly) measure the same construct. This is pretty straightforward when looking at the items concerning suicidal ideations in both MD and BPD. Secondly, this research used self-report to measure the symptoms of BPD and MD. This means that it is unclear whether the responses are accurate reflections of the presence of symptoms. However, all patients received a DSM diagnosis from their primary practitioner which showed that at least 21% of patients suffered from (traits) of BPD and 28% of patients suffered from some kind of mood disorder. Another limitation of this study is the operationalization of the symptoms of BPD and MD. The number of nodes had to be limited in an attempt to make a reliable estimation of the comorbidity network. The BDI-II contains 21 items that measure the 10 symptoms of depression as included in the DSM. As argued above, we only used the BDI-II items that matched best with the DSM-criteria of MD. The SCID-II contains fifteen questions to measure the nine symptoms of BPD as included in the DSM. As described above, although most questions in the SCID-II have a one-on-one relationship with a DSM criterion of BPD, there are three BPD criteria (“identity diffusion”, “anger”, “self-harm/suicidality”) that are measured by multiple questions in the SCID-II. For these three criteria we decided only one question needs to be answered positive to score the criterion. This operationalization may have had an influence on the variation of these three symptoms (less variation). Nevertheless, since the concerning criteria do not have a central role in the comorbidity network, this may not be considered a problem.

It is important to further test our hypothesis and replicate current findings in other and larger clinical samples, as to overcome the methodological limitations regarding the stability and accuracy of our empirically derived MD-BPD network model. In addition, it would also be interesting to look at gender differences in larger and other clinical samples to be able to compare the MD-BPD network model of females and males. Although the MD-BPD network may not be conclusive or factual, this exemplification does illustrate an alternative perspective on the comorbidity of personality disorders and mental disorders. This contribution harmonizes with a systemic or relationalist ontology when viewing and understanding mental disorder. Instead of conceptualizing and studying particular symptoms and/or syndromes as isolated diseases that reflect underlying disease causes, a systemic or relationalist view entails that we understand psychiatric symptoms and/or syndromes as existing and functioning in relationship to each other. We cannot view mental disorders and personality disorders in isolation; the particulars that constitute the reality of mental disorder only exist and have meaning in their relationality (see also Köhne, 2020).

## Conclusion

This contribution emphasizes the importance of embracing novel methodological and theoretical frameworks in the field of comorbidity research and brings an exemplification of this in the form of the first network-analysis of the links between symptoms of MD and BPD. If the symptoms of mental disorders and personality disorders are indeed meaningfully connected, it may be relevant to identify and target the most important links therapeutically by which we may prevent this type of comorbidity in which one disorder activates the next disorder. For the field to progress, we argue that embracing a thinking frame that more adequately and accurately approximates the complexity of the human mind is imperative. It is thus important to look for new building blocks and ways to meaningfully integrate the interacting layers of the neuro-biological, psycho-experiential and socio-cultural when understanding the causes and mechanisms of mental disorder. Symptoms are embedded in persons with bodies that are embedded in communities, societies and histories.

## Data Availability Statement

The original contributions presented in the study are included in the article/Supplementary Material, further inquiries can be directed to the corresponding author.

## Ethics Statement

The study protocol was approved by the institutional review board of GGZ Momentum and was performed in accordance with the Netherlands Code of Conduct for Research Integrity and was carried out in accordance with The Code of Ethics of the World Medical Association (Declaration of Helsinki) for experiments involving humans. All subjects provided written informed consent.

## Author Contributions

AK developed the study concept. All authors contributed to the study design. Data collection was performed by AK. All authors performed the data analysis, interpretation and critically revised all versions of the paper. All authors approved the final version of the paper for submission.

## Conflict of Interest

The authors declare that the research was conducted in the absence of any commercial or financial relationships that could be construed as a potential conflict of interest.
