# Supplementary material for: A Network Perspective on the Comorbidity of Personality Disorders and Mental Disorders: An Illustration of Depression and Borderline Personality Disorder
Source: Front Psychol. 2021 Jul 6;12:680805. doi: 10.3389/fpsyg.2021.680805 (PMC8290338; doi:10.3389/fpsyg.2021.680805)
Supplement: Supplementary file 1 [file Data_Sheet_1.pdf]

Supplementary Materials

Supplementary Figure 1: Accuracy of the edge-weights

Supplementary Figure 2: Bootstrapped significance difference test for edge weights

Supplementary Figure 3: MD-BPD network using Spearman correlations

The authors have provided this supplementary material to give readers additional information about the accuracy and stability of the Major Depression (MD) and Borderline Personality Disorder (BPD) network. Using the R-package *bootnet*[†] version 1.4. we checked whether the estimated network associations were stable and accurate. Supplementary Figures 1 and 2 below show the bootstrap results, based on 1000 iterations.

[†]Epskamp, S., Borsboom, D., & Fried, E. I. (2018). Estimating psychological networks and their accuracy: A tutorial paper. *Behavior Research Methods*, *50*(1), 195–212. https://doi.org/10.3758/s13428-017-0862-1

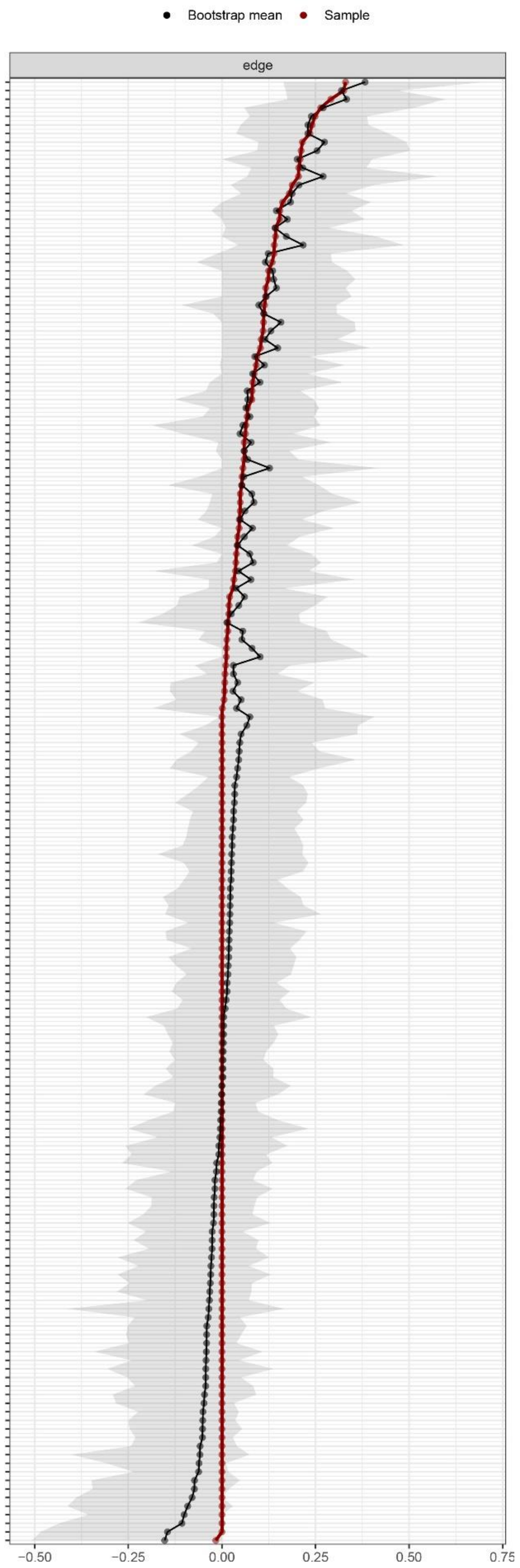

Supplementary Figure 1. Accuracy of the edge-weights. On the y-axis are the edges of the MD-BPD network (the y-axis labels have been removed to avoid cluttering), ordered from the highest edge (top) to the lowest edge (bottom). The red dotted line indicates the sample values for the analyzed data, while the black dotted line indicates the bootstrap mean values. The gray area represents the bootstrapped CIs. The sample values lie within the bootstrapped confidence intervals and the bootstrap mean values are generally aligned with the sample values. However, and of note, the bootstrapped CIs are very wide, ranging from positive to negative values for many of the edges, and thus caution is recommended especially when interpreting the presence and strength of weaker edges.

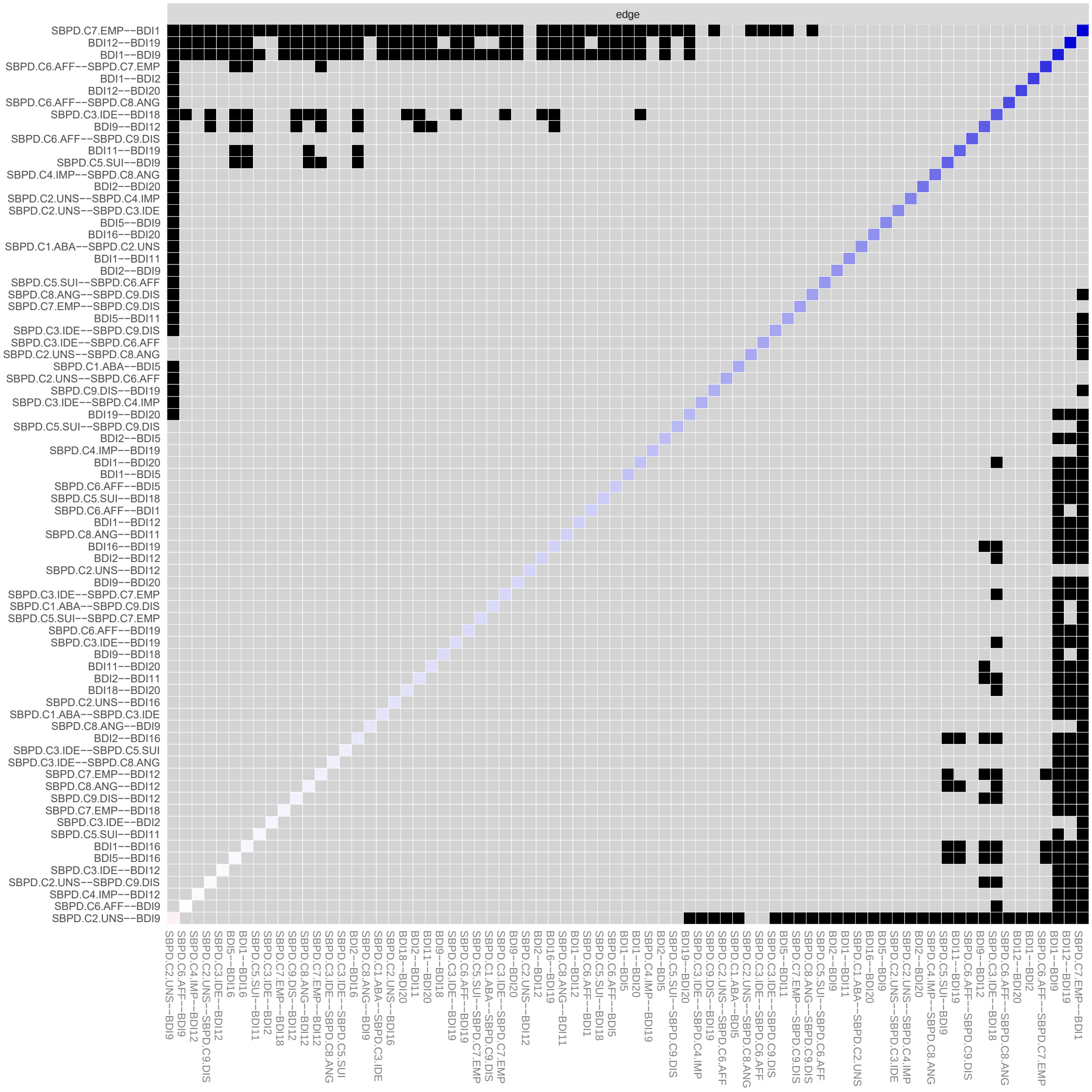

Supplementary Figure 2. Bootstrapped difference test for the edge weights of the MD-BPD network. The significance difference testing (α=0.05) explores whether each edge in the network is significantly larger than the other edges in the network. The color of the boxes indicates whether there is a significant difference (i.e., black boxes reflect significant differences, gray boxes reflect no significant differences). The diagonal line indicates the strength of edge-weights, shifting from white (representing weaker edges) to blue (representing stronger edge-weights). In this case only a few edges significantly differ from the other edges in the network, though not always from each other.

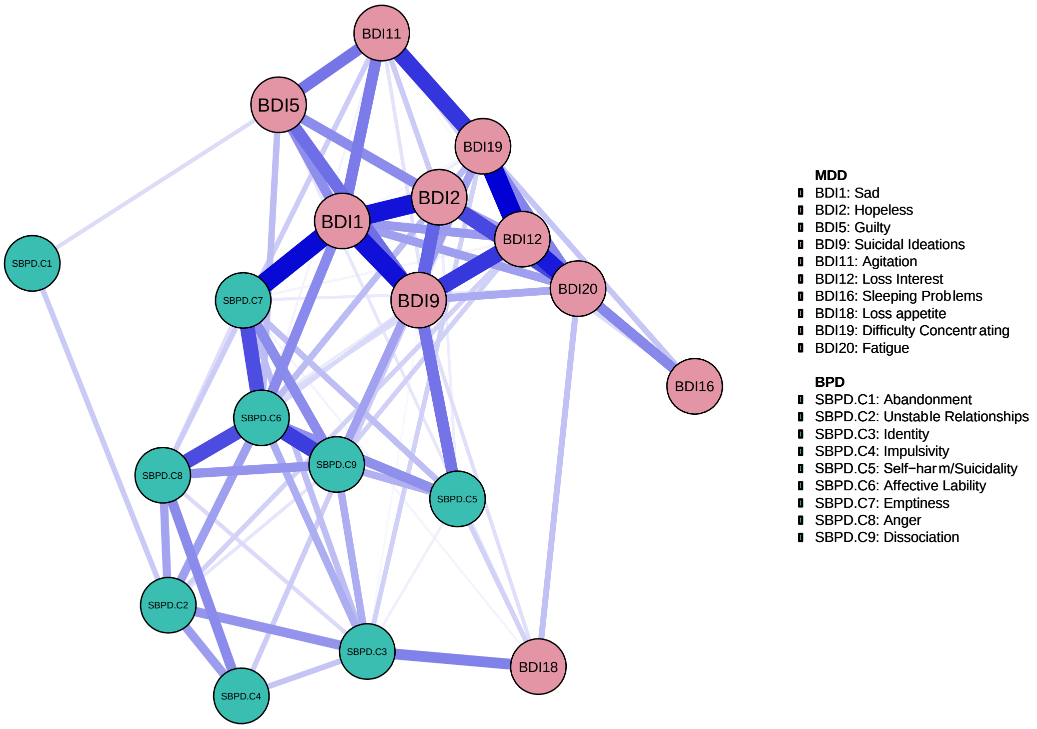

Supplementary Figure 3. MD-BPD network using Spearman correlations as input. The main findings are well-aligned with the findings presented in the manuscript; the network structure retrieved, while having a different layout, displays a high similarity to the original network structure described in the manuscript.
